# Advancing the strategic use of HIV operations research to strengthen local policies and programmes: the Research to Prevention Project

**DOI:** 10.7448/IAS.18.1.20029

**Published:** 2015-08-18

**Authors:** Deanna Kerrigan, Caitlin E Kennedy, Alison Surdo Cheng, Sarah J Sandison, Virginia A Fonner, David R Holtgrave, Heena Brahmbhatt

**Affiliations:** 1 Department of Health, Behavior and Society, Johns Hopkins Bloomberg School of Public Health, Baltimore, MD, USA; 2 Department of International Health, Johns Hopkins Bloomberg School of Public Health, Baltimore, MD, USA;; 3 US Agency for International Development, Washington, DC, USA; 4 Department of Population, Family, and Reproductive Health, Johns Hopkins Bloomberg School of Public Health, Baltimore, MD, USA

**Keywords:** operations research, implementation science, HIV, prevention, USAID

## Abstract

In the field of HIV prevention, there is renewed interest in operations research (OR) within an implementation science framework. The ultimate goal of such studies is to generate new knowledge that can inform local programmes and policies, thus improving access, quality, efficiency and effectiveness. Using four case studies from the USAID-funded Research to Prevention (R2P) project, we highlight the strategic use of OR and the impact it can have on shaping the focus and content of HIV prevention programming across geographic and epidemic settings and populations. These case studies, which include experiences from several sub-Saharan African countries and the Caribbean, emphasize four unique ways that R2P projects utilized OR to stimulate change in a given context, including: (1) translating findings from clinical trials to real-world settings; (2) adapting promising structural interventions to a new context; (3) tailoring effective interventions to underserved populations; and (4) prioritizing key populations within a national response to HIV. Carefully crafted OR can bridge the common gap that exists between research-generated knowledge and field-based practice, lead to substantial, real-world changes in national policies and programmes, and strengthen local organizations and the use of data to be more responsive to a given topic or population, ultimately supporting a locally tailored HIV response.

## Introduction

Although not a new concept, operations research (OR) is an area of renewed interest to public health policymakers and programme planners [1]. OR approaches have gained greater recognition in the field of HIV in recent years, particularly as they seek to understand how to maximize the effectiveness of HIV prevention interventions [2–5]. The 2013 Evaluation of the President's Emergency Plan for AIDS Relief (PEPFAR) conducted by the Institute of Medicine specifically recommends that HIV programming be informed by a prioritized OR portfolio that optimizes the effectiveness, quality and efficiency of services and also contributes to the global knowledge base on implementation of HIV programmes [6]. PEPFAR situates OR within a broader implementation science framework, where routine monitoring and evaluation, OR studies and focused impact evaluations all work together to provide critical data to public health decision makers regarding the development and refinement of the most strategic HIV response within a given context [7].

OR has been defined as a continuous process with five basic steps: (1) problem identification and diagnosis; (2) strategy selection; (3) strategy testing and evaluation; (4) information dissemination; and (5) information utilization. The goal of OR is generally understood as generating evidence that can assist to increase the efficiency, effectiveness and quality of services delivered by providers, as well as the availability, accessibility and acceptability of services for users (Population Council, HIV OR Handbook; WHO OR guidance) [8, 9]. More recently, investigators have also highlighted the importance of using OR to find innovative ways of maximizing scarce resources and reducing programme costs while maintaining quality [10, 11]. In essence, OR creates new scientific knowledge that policy and programme planners can use to improve investment options, including data that help to assess the feasibility of new intervention strategies; determine how to implement interventions in new settings, populations or at scale; or advocate for changes to policy and practice.

The growing interest in OR has been stimulated in part by recent biomedical advances in the field of HIV prevention and the increased emphasis on combination HIV prevention approaches [12]. For example, there is now a range of established biomedical approaches to HIV prevention including Treatment as Prevention (TasP), Prevention of Mother-to-Child HIV Transmission, Voluntary Medical Male Circumcision (VMMC), Post-Exposure Prophylaxis, and Pre-Exposure Prophylaxis. However, formidable gaps exist between knowing what works in focused, controlled trial settings and creating and sustaining effective programmes at scale in the real world [3, 10, 11, 13]. In turn, important gaps in understanding remain regarding how to best maximize the effectiveness of such interventions, as well as how to strategically integrate behavioural and structural prevention interventions and measure their unique and synergistic contribution to HIV outcomes within a comprehensive prevention package.

Although many key OR questions have global relevance, ultimately, OR seeks to improve HIV outcomes at the local level. Previous work has identified the central role of context in OR, including the sociocultural, politico-legal and economic aspects of a setting, as well as specifics of a given health system and, in the case of HIV, dynamics of the local epidemic [14]. To effectively understand and address such contextual issues, collaboration and partnership are often critical components of the OR process so that OR studies are asking questions that are grounded in local realities and priorities, and ensuring the strategic use and sustainable uptake of research findings in practice [15, 16].

Research to Prevention (R2P) was a USAID-funded task order conducted between 2008 and 2014 under the broader Project SEARCH (Supporting Evaluation and Research to Combat HIV/AIDS) OR umbrella. R2P aimed to identify critical knowledge gaps and inform programme strategies for effective HIV prevention including improving the quality and coverage of prevention interventions in countries most affected by the epidemic, particularly sub-Saharan Africa. R2P had three key objectives: (1) to conduct OR in the area of HIV prevention; (2) to promote dissemination and utilization of data generated in the OR process; and (3) to build the capacity of local researchers and governmental and non-governmental institutions to conduct and use research findings.

R2P conducted approximately 30 HIV prevention OR activities across 18 countries. In each of its field-based projects, collaboration with local research partners and engagement with government institutions was prioritized to conceptualize and implement a given activity and for capacity building. As a result, findings from R2P studies contributed to the addressing challenges that were relevant to the global OR agenda but were equally resonant and appropriate for the local context. Although R2P projects sometimes focused on a specific previously established biomedical, behavioural or structural intervention strategy, the research was situated in the socio-political context of a given setting.

Here, we highlight examples of R2P OR projects across a variety of geographic and epidemic settings and population groups. Through each of the four case studies presented, we demonstrate how context-specific OR questions implemented collaboratively can lead to the prioritization and improvement of local HIV prevention programmes and policies. These cases emphasize several ways that OR can be strategically used to shape better HIV prevention decision making and outcomes, including: (1) translating findings from clinical trials to real-world settings; (2) adapting promising structural interventions to a new context; (3) tailoring effective interventions to underserved populations; and (4) prioritizing key populations within a national response to HIV, extending the manner in which we understand the role and utility of OR.

## Translating the results of clinical trials into practice: scaling up VMMC programmes in sub-Saharan Africa

Three randomized controlled trials previously demonstrated the efficacy of VMMC in reducing HIV acquisition risk among men [17–19]. As a result, the World Health Organization recommended the implementation of VMMC as a key HIV prevention strategy in 2007. The trial findings led to rapid expansion of VMMC services by donors and governments in priority countries in eastern and southern Africa. However, questions arose regarding whether health systems in these settings would be able to create and meet the demand for VMMC while maintaining high-quality services. Countries have varied in their approaches to achieving VMMC scale-up targets, including variability in surgical procedures utilized, types of personnel engaged and settings where VMMC services are delivered. The R2P project Systematic Monitoring of the Voluntary Medical Male Circumcision Scale up (SYMMACS) was a facility-based study conducted in four priority countries for VMMC scale-up – Kenya, South Africa, Tanzania and Zimbabwe – that sought to assess the efficiency and quality of VMMC surgical procedures during the scale-up process [20]. SYMMACS included quality assessments of each site, interviews with care providers, direct observations of VMMC procedures and compilation of VMMC clinic data. Two rounds of data were collected in 2011 and 2012.

Results from SYMMACS showed that safe, high-quality VMMC can be implemented and sustained at scale, although quality was found to decline in areas where the number of new VMMC sites increased rapidly [21, 22]. Infection control, pre-operative examinations and post-operative patient monitoring and counselling were identified as areas for improvement and future monitoring, though specific findings differed by country. The adoption of practices designed to improve surgical efficiency in the operating room also varied by country, reflecting national policies. For example, at the time of the study, Kenya and Tanzania effectively employed task shifting by allowing nurses to perform the VMMC procedure in place of physicians, but this practice was not allowed in South Africa and Zimbabwe. SYMMACS data confirmed the efficiency benefits of task sharing of suturing and the use of electrocautery for decreasing total operating time without decreasing the quality of care [22]. SYMMACS also identified the factors associated with provider attitudes towards VMMC efficiency elements [23] and provider burnout [24].

The SYMMACS study team engaged in intensive data dissemination efforts in each study country and globally to extend the reach and utilization of the findings. This included conducting data utilization workshops, presenting findings to policymakers and collaborating with local investigators to publish results in peer-reviewed journals. Notably, SYMMACS results contributed to a change in government policy in Zimbabwe, which began allowing task shifting of VMMC procedures from doctors to nurses as of January 2014 [25]. Elements of SYMMACS monitoring have also been incorporated into routine programme monitoring in South Africa [26]. Through two years of targeted data collection observing clinics and interviewing providers, SYMMACS helped to focus greater attention on the need for ongoing systematic assessments of the efficiency and quality of VMMC services across settings as scale-up of this intervention continues to expand.

## Adapting promising structural HIV prevention interventions in new contexts: cash transfer initiatives for disadvantaged youth in urban South Africa

The high burden of HIV and sexually transmitted infections (STIs) in South Africa, especially among young people in disadvantaged urban settings, has led to increased interest in interventions that address the underlying structural drivers of the epidemic. Cash transfer interventions that provide monetary incentives to participants aim to address the socioeconomic context of HIV infection such as poverty [27]. To date, most cash transfer studies have been conducted among young women in rural African settings with the primary goal of improved school attendance [27, 28]. Given the rapid pace of urbanization in lower and middle income countries and the “urban penalty,” which includes high rates of unemployment, migration, unstable housing, increased alcohol and drug use, violence, HIV and other STIs, understanding the feasibility and acceptability of a cash transfer intervention in these settings is of particular importance. The R2P study CHANGE was a randomized comparative trial of 120 young females and males (16–18 years old) examining the socio-economic and HIV risk profiles of young people in inner city Johannesburg, South Africa, the pathways by which lack of access to resources leads to HIV risk and the potential for economic interventions to mitigate this risk [29]. Three cash transfer strategies were evaluated: (1) an unconditional monthly cash transfer over six months; (2) a monthly cash transfer conditioned on school attendance over six months; or (3) a single cash transfer conditioned on attendance at a youth-friendly clinic for a sexual and reproductive health visit.

At six-month follow-up, the CHANGE study found that the clinic condition arm demonstrated a significant impact on health behaviour, with three times more clinic visits occurring in this group compared to other study arms [29]. This finding is particularly salient in light of increased emphasis in the field on service use uptake, including HIV counselling and testing, and linkages to care as part of treatment or TasP. Results demonstrated high feasibility and acceptability of cash transfers in an urban setting where adolescents themselves receive transfers, with little evidence of unintended negative consequences such as spending money on alcohol or drugs. The South African government has a child's social grants programme in place for economically vulnerable families with children less than 18 years of age [30–32]. Findings from this R2P study offer critical information to this government programme on the conditioning of social grants and their potential impact in reducing the structural HIV risk of disadvantaged urban youth.

## Tailoring HIV prevention and care services for key populations: a multilevel intervention among female sex workers living with HIV in the Dominican Republic

Female sex workers (FSW) have a significantly higher burden of HIV infection than adult women in the general population across settings [33]. Yet, few interventions exist to address the needs of FSW living with HIV including specific barriers to engagement in HIV prevention, treatment and care services. To address this gap, formative research conducted under R2P was utilized to develop a multilevel, community-based intervention for FSW living with HIV called *Abriendo Puertas* or “Opening Doors.” *Abriendo Puertas* was implemented collaboratively by local research, non-governmental organizations (NGO) and community partners, including a sex-worker led group. The intervention sought to address HIV and sex-work-related stigma and discrimination experienced by FSW living with HIV in multiple spheres of their lives including their sexual relationships, work, HIV clinics and communities [34]. The approach included a combination of individual counselling and health education; peer HIV service navigation; sensitivity training of HIV clinical care providers; community solidarity and mobilization activities; and the offering of HIV testing and counselling, and linkages to care for male partners. A cohort of 250 women was enrolled to assess the feasibility of the intervention and its initial effects on prevention, treatment and care outcomes. Mixed methods including structured surveys, biological assessments of STIs and viral load, and qualitative research were utilized to understand the process and impact of the intervention over time.

After 10 months, women in the *Abriendo Puertas* intervention showed statistically significant improvements in consistent condom use with all sexual partners in the last month [35]. Engagement in HIV care services, treatment continuity and adherence to anti-retroviral therapy also improved significantly among participants. In addition, approximately one-fourth of participating women referred their male partners for HIV testing and counselling during the intervention, with seven new cases of HIV detected and nine men linked to care. Both female and male participants found the intervention highly acceptable and empowering. Findings suggest that by addressing population and setting specific aspects of living with HIV among this group, including social and structural factors, uptake of HIV services and key prevention and care outcomes can be improved. The study team engaged local policymakers and communities in data utilization efforts, producing a video and holding a workshop to discuss next steps. With strong community and government support for the intervention, the model is now being integrated into the local HIV care system with USAID support and extended to other key populations including men who have sex with men (MSM) and transgender women [36, 37].

## Prioritizing the needs of underserved groups: using data to expand the scope of the national HIV response to include key populations in Swaziland

Planning an effective national HIV response requires knowing the local epidemiology of HIV and the social and institutional factors that may affect the success of different intervention strategies. In Swaziland, key populations such as MSM and FSW had never been the focus of rigorous behavioural and biological surveillance prior to this R2P project, although these populations were known to be at heightened risk of HIV in other settings, even in generalized HIV epidemics [33, 38]. The 2009–2014 Swaziland National Multi-Sectoral Strategic Framework for HIV and AIDS [39] noted that “Quality and reliable data are largely lacking for most of the key populations,” and attributed this gap to “lack of prioritization of such populations in prevention programmes.” R2P collaborated with the Swaziland Ministry of Health and other NGOs to conduct a quantitative survey using respondent-driven sampling to estimate population-level prevalence of HIV and risk factors among MSM and FSW in Swaziland [40–46]. The project also included complementary qualitative research including in-depth interviews and focus group discussions among MSM and FSW living with HIV to understand their experiences and unique needs in prevention, care and treatment services [47, 48]. In this study, HIV prevalence was estimated at 60.5% among FSW and 12.6% for MSM [40, 49]. Both populations reported experiencing significant stigma, discrimination and structural barriers to successfully engaging in HIV prevention, care and treatment services [43, 48].

Findings from the study enabled the Swaziland Ministry of Health to better focus their HIV response to their local epidemic and social, cultural and institutional context. For example, fear of stigma, violence and harassment from police and healthcare institutions were identified as barriers to uptake of HIV services, so current interventions include key populations sensitivity training with the Royal Swazi Police, adaptations to make clinics friendlier to key populations and community dialogues between MSM, FSW and healthcare providers [50]. In addition, the R2P project helped to engage key populations in the national HIV response, including an application to the Global Fund [51]. As a result of study findings, the Swaziland government has made plans to fund HIV-related projects for key populations [52] and has invited representatives from the MSM and FSW communities to engage with national technical working groups on HIV prevention [48]. Study findings also shaped the Swaziland Ministry of Health's policies towards engaging these key populations, which had previously received only minimal attention [39]. Funding from international donors for HIV prevention activities among key populations has also increased since this study took place [50]. In addition, these efforts helped to catalyse the formation of a sexual minority rights organization, which is in the process of utilizing R2P study findings to develop a training manual to sensitize HIV clinical care providers to better serve the needs of key populations, through a subsequent R2P OR local dissemination and utilization grant [53].

## Implications for future HIV OR

These diverse case studies from R2P projects highlight how OR can be used to shape HIV prevention policy and programmes. These studies spanned a variety of uses of OR, including translating global clinical trial findings to “real world” settings, adapting promising interventions in new contexts, tailoring the implementation of interventions to increase uptake among underserved groups and using data to prioritize the inclusion of specific groups within a national HIV response. The cases also highlight the range of OR questions, study designs and methods that can be used to address implementation challenges across different HIV prevention interventions, geographic and epidemic settings, and population groups. The examples emphasize the importance of collaboratively crafting strategic, context-specific OR to address the complex pathways of HIV risk and service uptake in different settings.

The SYMMACS study showed how a key finding from randomized controlled trials was translated into real-world programming, and how OR can help document this transition, assess whether quality and efficiency of services are maintained during national scale-up in different countries and inform policy decisions for national programmes. This is exactly the “know–do” gap that OR is designed to fill. The CHANGE study demonstrated how OR can be used to adapt promising interventions in a new context, providing policymakers and programme planners with important information about how social, economic and structural differences may shape the pathways through which cash transfers affect HIV-related risk and service use behaviours among at risk youth in urban, resource-poor settings. *Abriendo Puertas* showed how formative research findings can be an important component of OR and used to tailor a multilevel intervention model for a specific population. Finally, the R2P project, with key populations in Swaziland, provided data that helped broaden the scope of a national HIV response while also empowering community members to take an active role. Advocating for policy change is one of the primary purposes of OR [54], and OR findings should help inform resource allocation and policy decisions [11]. Furthermore, engaging community groups in research is one way to bring them into the HIV response and facilitate capacity building, ownership of findings and empowerment. In summary, these case studies highlight the multiple phases and types of research methods and study designs that OR can use to monitor and improve existing programmes, identify underserved populations in need of interventions, increase uptake of interventions, understand the pathways through which interventions work and translate effective interventions to novel contexts.

At the local level, each of the four R2P case studies shows how OR can be an integral component of informing policies and programmes (such as VMMC programmes), developing tailored programmes (such as *Abriendo Puertas*), assessing the feasibility of interventions to inform ongoing policy debates (such as the CHANGE study) and providing information to inform local HIV programming and investment decisions (such as current programming for key populations in Swaziland). These case studies also show how OR can directly affect local policies in a governmental health system, such as Zimbabwe's new national policy to allow nurses to provide VMMC or Swaziland's decision to consult key populations when developing national proposals concerning HIV prevention, care and treatment [25, 51]. Finally, they show how OR can be used to strengthen capacity among local organizations, including governmental and civil society organizations of vulnerable groups.

Although R2P OR studies generated knowledge that had local impact, the studies also had global relevance. The findings from SYMMACS will be used to inform the scale-up of VMMC services in other high-priority countries in sub-Saharan Africa, and the findings from the CHANGE study will be added to the currently limited global evidence base on the pathways through which cash transfers work to change behaviour and the intended and unintended consequences of an economic intervention in an urban, disadvantaged setting that targeted both females and males. Furthermore, models used for the OR in R2P can serve as a template for other work in OR. For example, as countries increasingly recognize the importance of having reliable data on key populations, the idea of using data to advocate for including underserved populations in the national HIV response could be replicated elsewhere. The methodologies used in R2P can be replicated as well. For example, the formative work used to design *Abriendo Puertas* could be used to identify and tailor feasible and empowering interventions for sex worker populations in different contexts.

Globally, the HIV response is currently characterized by a period of transitions to increasingly country-led initiatives and calls for increased programme efficiencies [55]. In the context of these policy shifts and economic realities, OR will continue to play an important and potentially larger role in shaping HIV programmes and policies. OR will be essential to prioritize and assess the contributions and pathways of unique and novel HIV prevention approaches for which there is comparatively less evidence, particularly for structural and behavioural interventions. The approaches most likely to be successful will draw on diverse disciplines to elevate the level of *status quo* thinking that has, to date, not adequately advanced the field of HIV prevention to appropriately tailor effective interventions to diverse contexts. For example, better aligning HIV prevention interventions with socio-cultural and political contexts may be critical to narrowing the translational gap to effective prevention programmes [56]. Achieving viable programmes that integrate HIV services, including prevention efforts, into broader public health systems and international social and economic development objectives also requires OR that is focused on optimizing sustainable and scalable systems.

We see several opportunities for improving the OR agenda in the coming decade. First, there is a need to better integrate routine monitoring and evaluation with OR, linking routine daily assessment of inputs and outputs with OR questions of efficiency and effectiveness [7, 57]. Second, researchers can make better use of existing programme data via secondary analyses to inform interventions. Third, R2P's mandate included incorporating capacity building and dissemination of findings for every research project and activity. We emphasize the need to build capacity so programme staff can be empowered to recognize obstacles/bottlenecks, have mechanisms in place to research problems and identify/implement solutions, and elevate the capacity of local institutions and researchers to lead future work [58, 59]. In many R2P projects, local and national governments and community-based organizations were meaningfully engaged in capacity building efforts. Because these groups had in-depth knowledge of local information needs and priorities, they were best placed to engage communities, ask relevant questions and drive the process of data dissemination and utilization of R2P study results. This led to a substantive difference in data utilization and impact on programmes. As there is greater recognition of the role of dissemination and translation of OR findings into usable results for programme planners and policymakers, funding for OR should continue to include technical support and funds for dissemination and data utilization activities with an emphasis on incorporating these components from the outset and throughout the OR process [8, 60].

Finally, as PEPFAR moves towards more sustainable programmes and HIV epidemic control by targeting geographic areas and populations most at need with evidence-based, high-impact interventions [55], there will be an increasing need for high-quality OR that can address questions of efficiency and effectiveness of these efforts and provide data for decision making.

## Conclusions

We present four case studies from the USAID-funded R2P project demonstrating how the strategic use of OR can make specific impacts at multiple levels, including how it can lead to substantial changes in local programmes, national policies and global agendas, and how it can have an impact on the development of organizations and sustainable responses, tailoring the content of interventions for local contexts and population groups. Together, these examples highlight how we can contextualize a global OR and implementation science agenda, bring studies and their findings into national dialogues and policy debates, and partner effectively with local groups to make meaningful social change, strengthen the HIV response and positively have an impact on the people most affected by HIV.
